# Data Collection and Remote Control of an IoT Electronic Nose Using Web Services and the MQTT Protocol

**DOI:** 10.3390/s25144356

**Published:** 2025-07-11

**Authors:** Juan J. Pérez-Solano, Antonio Ruiz-Canales

**Affiliations:** 1 Departamento de Informática, Universitat de València, Avenida de la Universidad S/N, 46100 Burjassot, Valencia, Spain; 2 Departamento de Ingeniería, Universidad Miguel Hernández, Campus de Orihuela, Carretera de Beniel, 03312 Orihuela, Alicante, Spain; acanales@umh.es

**Keywords:** electronic nose, web-services, VPN, Python

## Abstract

An electronic nose is a device capable of characterizing samples of substances and products by their aroma. The development of such devices relies on a series of non-specific sensors that react to gases and generate different signals, which can be used for compound identification and sample classification. The deployment of such devices often requires the possibility of having remote access over the Internet to manage their operation and to collect the sampled data. In this context, the application of web technologies to the monitoring and supervision of these systems connected to the Internet, which can be considered as an Internet of Things (IoT) device, offers the advantage of not requiring the development of client-side applications. Users can employ a browser to connect to the IoT device and monitor or control its operation. Moreover, web design enables the development of cross-platform web monitoring systems. In addition, the inclusion of the MQTT protocol and the utilization of a virtual private network (VPN) enable a secure transmission and collection of the sampled data. In this work, all these technologies have been applied in the development of a system to manage and collect data to monitor rot in lemons treated with sodium benzoate before harvest.

## 1. Introduction

Electronic nose (eNose) devices are essential for characterizing samples based on their aroma, offering an alternative to traditional sensory methods and gas chromatography techniques. eNose technology utilizes a series of non-specific sensors that react to gases, generating various signals. These signals, once processed for feature extraction, can be used for compound identification and sample classification based on the emitted aromas. eNose devices usually consist of non-specific resistive sensors that respond to gases and produce signals. They can be used to classify gaseous samples depending on their volatile compounds, creating a combined sensor response that forms an odor pattern. In this paper, an eNose prototype with 8 MQ metal oxide sensors was connected to the Internet to enable secure remote management of its operation and to perform the data collection process using web services. This eNose [1] can characterize and classify different agrifood products through the information collected. The proposed architecture allows a secure connection of an eNose to the Internet, which, with this scheme, can be considered an Internet of Things device [2] integrated into the web [3].

The relevance and feasibility of implementing IoT-enabled eNose systems in practical scenarios is highly dependent on the specific application domain and the associated connectivity constraints. Thus, in remote agricultural monitoring, global Internet connectivity is essential for aggregating data from distributed farming operations and enabling centralized decision-making, such as the early detection of crop diseases or fruit spoilage [4]. Additionally, postharvest logistics benefits from real-time eNose monitoring of perishable goods during transportation, which relies on continuous Internet access to support data analytics and alert systems that ensure timely interventions and reduce losses.

The connectivity of IoT devices through the web enables real-time remote monitoring and control of these devices to improve production processes, increasing efficiency and accessibility to information. In agriculture, IoT via web technology facilitates efficient resource management, task automation and fault prediction, reducing production costs and waste of resources [5]. In addition, the collection and analysis of data generated by connected devices provides valuable information for decision-making and process optimization. The integration of IoT with the web is transforming the way we interact with our environment, driving efficiency, convenience and sustainable development [6].

The development of web applications for the control of IoT systems offers numerous advantages over other alternatives. First, web applications are multiplatform, which means that they can be accessed from any device with a web browser, eliminating the need to develop specific versions for different operating systems. Updates and upgrades are implemented centrally on the server, ensuring that all users always have access to the latest version without the need to download and install updates. They also offer easier integration with other technologies and services through APIs, which improves functionality and interoperability. Security is another advantage, as data can be stored and protected on centralized servers, allowing for more controlled security management. All these advantages make web applications a highly efficient and flexible option to meet the needs of users and enterprises in the IoT device control environment [7].

The Message Queuing Telemetry Transport (MQTT) protocol [8] is commonly used to transport data from IoT devices to the server. This lightweight messaging protocol is designed for efficient communication between devices in a network. It is widely used in IoT systems and other cases where low power consumption, low bandwidth usage and reliable messaging are crucial. In essence, MQTT follows a simple publish-subscribe model, where devices/applications can publish messages to a specific topic, and other devices or applications can subscribe to those topics to receive the messages. This decoupled nature allows for scalable and flexible communication between multiple devices without direct point-to-point connections. One of the main features of MQTT is its lightweight nature. The protocol is designed to be extremely efficient and requires minimal bandwidth and resources. This makes it ideal for environments with devices with limited processing power and memory. MQTT also supports Quality of Service (QoS) levels, which determine the reliability and message delivery guarantees. Three QoS levels are available:

- QoS 0 (At most once): At this level, messages are delivered on a best-effort approach. They may be lost or delivered multiple times. It provides the lowest level of reliability.
- QoS 1 (At least once): With QoS 1, messages are guaranteed to be delivered at least once but may be delivered multiple times. This level guarantees the reliability of message delivery, although duplicates may occur.
- QoS 2 (Exactly once): QoS 2 provides the highest level of reliability. Messages are delivered once and only once. At this level, a handshake protocol is used to confirm message delivery and eliminate duplicates.

This flexibility allows developers to choose the appropriate QoS level based on their application requirements. For example, applications that prioritize speed and efficiency over message reliability can opt for QoS 0, while those that require strict message delivery guarantees can use QoS 2. In addition, MQTT is designed to be platform-independent, so it is compatible with a wide range of programming languages and operating systems. There are MQTT client libraries available for popular programming languages such as Python, Java, JavaScript, etc., making it easy to integrate MQTT into any application. In our case, we have chosen Mosquitto broker [9], also known as Eclipse Mosquitto, which is a popular and widely used open-source MQTT broker. Mosquitto broker is known for its simplicity, reliability and ease of use, and acts as a backbone to facilitate communication between devices or applications in a distributed system.

As a conclusion, MQTT is a lightweight protocol with a low memory footprint and an efficient publish/subscribe architecture, making it particularly suitable for real-time, asynchronous communication in resource-constrained IoT environments such as agricultural deployments over cellular networks. Compared to HTTP’s request-response model and CoAP’s UDP-based design, MQTT offers superior scalability and delivery guarantees through configurable Quality of Service (QoS) levels, providing a robust and flexible communication layer for our system [10].

**Figure 1:**
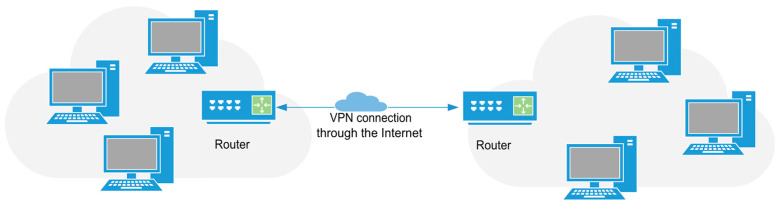
Site-to-site VPN connection.

The security of the connection to the IoT elements is essential when we are going to transmit information and configure the devices remotely through web interfaces. Securing IoT devices using VPN (Virtual Private Network) [11] is an effective strategy to protect data and communications in the era of massive connectivity. A VPN creates an encrypted tunnel through the public network, ensuring that data transmitted between IoT devices and servers is protected against interception and unauthorized access. Encryption processes based on Data Encryption Standard (DES), Triple DES (3DES) or Advanced Encryption Standard (AES) [12] standards are used to guarantee the confidentiality of data transmitted through this medium. This is crucial, given that many IoT devices operate with limited resources and may lack robust security capabilities on their own. By using a VPN, you can ensure data confidentiality and integrity, prevent attacks, and provide secure access to IoT devices from remote locations. In addition, a VPN can hide the location and IP address of the device, making the attacks more difficult. As a conclusion, implementing VPNs provides an essential additional layer of security to protect IoT devices and the sensitive data they handle, enhancing trust and security in increasingly interconnected environments. As shown in Figure 1, the VPN technology allows two or more networks to be remotely connected to each other over the public Internet. This is achieved by means of a virtual communication tunnel, which allows the data traffic between the networks to be private and secure, so that they appear to be directly connected. Site-to-site VPN is used to establish secure and reliable communication between networks separated by large geographical distances.

**Figure 2:**
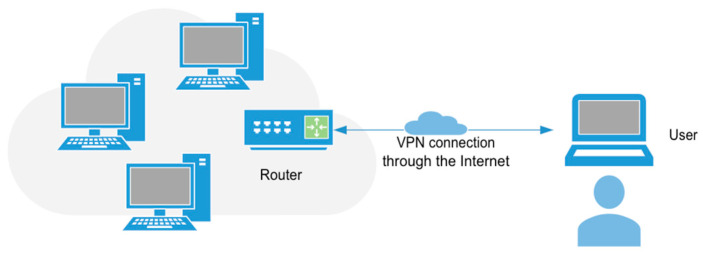
Client-to-site VPN connection.

But a VPN can also provide access to a company’s private network from anywhere via an Internet connection, as shown in Figure 2. In this case, after authentication on the network, the user, who may be referred to as a client, can work as if he were locally connected to the main network. To achieve this, the user must connect to a VPN server on the private network using client software that creates a virtual tunnel to the company, where a server, router or firewall is responsible for granting the connection provided that the user’s identity has been authenticated. In summary, the IoT device control and data collection system presented in this article enables the development of cross-platform applications, capable of controlling and accessing information from remote equipment in a secure manner.

While technologies such as MQTT, VPNs, and web-based interfaces are well-established and widely used in IoT systems, their integration into electronic nose (eNose) platforms for agricultural applications remains limited. Specifically, the development of secure, scalable, and resource-efficient architectures suitable for real-world deployment is still an open challenge. This is particularly relevant in agriculture and postharvest logistics, where early detection of spoilage, disease, and contamination is critical for minimizing losses and ensuring product quality.

To address these limitations, this work presents a modular, IoT-enabled eNose system designed for practical deployment in agricultural settings. The proposed architecture combines secure data transmission, real-time monitoring, and cross-platform accessibility through web services. Its capabilities are demonstrated through a comprehensive case study focused on the detection and monitoring of rot development in lemon fruits subjected to different pre-harvest treatments. This application not only validates the technical robustness of the system but also highlights its potential to support precision agriculture by enabling timely, non-invasive assessment of fruit quality under field-like conditions. Although initially developed for agricultural use, the system’s modular and adaptable design enables its application in other fields such as food safety, environmental monitoring and healthcare.

The rest of the paper is structured as follows. Section 2 is devoted to presenting the related work. In Section 3, a detailed explanation of the proposed system including all the software and hardware elements is provided. Section 4 comprises the experimental results obtained during the system evaluation. Finally, Section 5 includes the conclusions and discusses the contributions of the paper.

## 2. Related Work

Electronic noses based on metal-oxide sensors represent a rapidly advancing technology inspired by the human olfactory system, designed to detect, recognize, and differentiate volatile compounds in complex environments. These systems typically consist of arrays of metal-oxide semiconductor (MOS) sensors with nanostructured architectures, which can capture chemical information from the environment and translating it into digital signals through sophisticated pattern recognition algorithms. The unique properties of metal-oxide sensors such as high sensitivity, fast response, affordability and the ability to detect a wide range of volatile organic compounds (VOCs), make them particularly suitable for eNose applications across diverse fields. In healthcare, eNose are being developed as non-invasive, real-time diagnostic tools for disease detection through breath analysis, offering significant advantages over traditional methods by enabling quick, low-cost and preventive point-of-care diagnostics [13,14,15]. In the food and beverage industry, eNose are used for quality control, freshness assessment, and the detection of contaminants or adulteration in products such as meat, fish, grains, dairy, and beverages [16,17]. Agriculture also benefits from eNose technology, as these devices can provide early warning of crop diseases and pest infestations by detecting changes in emitted VOCs, thus supporting more sustainable and environmentally friendly farming practices [18,19,20]. Environmental monitoring is another key application area, where eNoses are employed to detect pollutants, control odors, and monitor air and water quality, with the ability to sense contaminants at very low concentrations [21,22,23]. Despite these advances, challenges remain, such as sensor drift, limited selectivity of individual sensors, and the need for robust data processing and standardization to ensure reliable operation in real-world conditions [13,24].

The electronic nose integrated into our IoT device was specifically designed for widespread use in the food industry and built using low-cost components. It features an array of eight MOS sensors, MQ-135, MQ-2, MQ-3, MQ-4, MQ-5, MQ-7, MQ-8, and MQ-9, capable of detecting a broad spectrum of VOCs. This configuration has proven effective not only in food quality monitoring but also in agricultural and environmental applications. A key innovation of our eNose is the application of a sinusoidal heating to the sensors. This technique involves varying the heater voltage in a sinusoidal pattern, which has been shown to improve the sensors’ sensitivity, selectivity, and resistance to drift. The sinusoidal heating allows the sensors to respond more dynamically to changes in the VOC environment, enhancing the overall accuracy of the device. By employing discrete Fourier transform (DFT) analysis on the sensor responses, the eNose can effectively discriminate between different varieties or qualities of the same product, thereby identifying fraudulent or misrepresented products before they reach consumers. This eNose has been successfully applied in the classification of olive oil [1], wine [25] and green coffee beans [26], as well as in the detection of pathogens like Colletotrichum coccodes in tomatoes [27]. Table 1 provides a summary of the applications of MOS-based eNoses, along with the corresponding references.

The combination of eNoses with the Internet of Things has allowed the autonomous operation of these devices in remote locations. The development of IoT-enabled electronic nose systems has seen significant advancements, particularly in applications related to environmental monitoring, food quality assessment, and medical diagnostics. For instance, an IoT-enabled eNose system has been developed for beef quality monitoring, utilizing sensors to detect spoilage through VOC concentrations, which are correlated with microbial growth [17]. Similarly, an IoT-enabled electronic nose has been applied in breath analysis, to classify and predict VOCs for potential diagnostic applications [15]. In environmental monitoring, eNoses have been employed for detecting airborne pollution hazards. These systems often use low-power wide-area network protocols like LoRa to facilitate remote monitoring without the need for constant internet connectivity [21,22].

Recent advancements on the Internet of Things and its integration with web technologies have significantly impacted various domains, particularly in building automation, smart cities, renewable energy, and agro-industrial systems. These studies highlight the diverse applications and potential of IoT technologies in enhancing energy efficiency and resources optimization. The combination of these technologies eliminates the need for client-side application design, allowing users to connect to the webserver via a standard browser to monitor and control IoT applications. Designing web interfaces coded in HTML (Hypertext Markup Language) [28], a cross-platform IoT system can be created, since HTML is compatible with browsers on a wide range of devices, including smartphones, computers, and tablets. This means that WEB-based IoT technology can enable remote cross-platform access, allowing administrators to control IoT systems using portable mobile devices. In this context [29], present a web-based control method applied in smart home technologies. A similar approach is proposed in the work [30] that proposes the development of a modular web-based interactive laboratory framework. The work in [31] shows the implementation of a remote-control system featuring a web-based graphical human–machine interface. Another example is the article of [32] that presents an interactive microgrid virtual laboratory focused on renewable energy education. This approach helps users engage with various controls and outcomes of the virtual grid, improving the understanding that these users have about grid systems. On the other hand, ref. [33] proposes the application of a web-based graphical interface to the optimization of robotic arm movements. Finally, the authors in [34] propose an IoT system for environmental and lighting control in buildings that integrates a web-interface based on node-RED. These studies collectively demonstrate the transformative potential of IoT across multiple domains. By addressing energy efficiency, environmental sustainability and resources optimization, these references provide a roadmap for leveraging IoT to meet contemporary challenges and enhance system capabilities.

However, the IoT connectivity introduces significant security and privacy challenges, since IoT devices present limited resources, making them vulnerable to attacks and data breaches [35]. The reliance on cloud-based services for data storage and processing further exacerbates these vulnerabilities, as it introduces additional security threats related to data privacy and secure communication [36]. In this context, Virtual Private Networks (VPNs) can be crucial for securing IoT systems. They establish encrypted tunnels between the system devices, safeguarding the network from eavesdropping and unauthorized access, thus ensuring data privacy. VPNs also enforce access control, allowing only authorized users or devices to interact with the IoT infrastructure through strong authentication mechanisms. Additionally, VPNs enable resource owners and administrators to securely access, manage, and maintain their devices remotely. Moreover, VPNs effectively combat Distributed Denial of Service (DDoS) attacks by channeling traffic through a centralized server, which filters and blocks malicious traffic efficiently [37].

In conclusion, although there are some previous studies that propose IoT-based systems for electronic nose data collection, they often do not consider key aspects such as secure communications, real-time interaction, and user accessibility. In particular, they do not incorporate web-based interfaces for direct user interaction, nor do they implement secure data transmission mechanisms suitable for deployment in open or remote environments. This work addresses these gaps by introducing a fully integrated, field-deployable IoT-eNose system with several novel contributions: (a) the design of a secure communication architecture using VPN and MQTT to ensure data integrity and confidentiality; (b) the development of a cross-platform web interface using Python and Flask, enabling intuitive remote control and real-time data visualization without the need for client-side applications; and (c) the validation of the system through a real-world deployment using cellular connectivity to monitor rot development in lemon fruits under different pre-harvest treatments. These contributions collectively demonstrate that the proposed IoT-eNose is a practical, scalable and secure solution capable of enabling real-time monitoring and supporting data-driven decision-making in agricultural applications.

## 3. Global System Architecture

**Figure 3:**
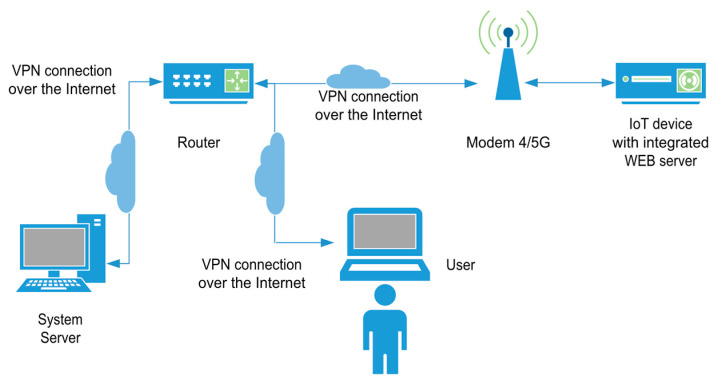
System architecture with an IoT-eNose, system server, and user. All of them are connected to the same VPN.

The system architecture, shown in Figure 3, consists of an IoT-enabled electronic nose, denoted as IoT-eNose, deployed in a remote location, and connected to the system via 4G/5G cellular modem that provide Internet access. A system server constitutes a hub for data storage, integrating a database and the MQTT broker. Users can interact with both the system server and the IoT-eNose. Through a web interface, users can access and configure the IoT-eNose, including setting up the data sampling process. Additionally, the system server offers a separate web interface that allows users to visualize the collected data efficiently.

The IoT-eNose integrates a Raspberry Pi 4 (Raspberry Pi Ltd., Cambridge, UK), running the Linux-based 64-bit Raspberry Pi Os, one eNose [1], and a 4G/5G cellular modem. The Raspberry Pi and the eNose are linked through a USB cable, enabling the exchange of data and commands between the two devices. Additionally, the Raspberry Pi is connected to the 4G/5G cellular modem via an Ethernet cable. In the system architecture, the IoT-eNose, the system server, and the user’s computer are interconnected within a secure VPN provided by the router. This secure communication setup relies on the router having a network interface with a static and public IP address. The Raspberry Pi executes an OpenVPN client to join the VPN. This OpenVPN client is installed with systemd support, and it automatically starts during the system boot using the configuration file that contains the certificates for the authentication process. Next, a detailed explanation of the development of the IoT-eNose and the system server is provided.

## 4. IoT-Enose Architecture

**Figure 4:**
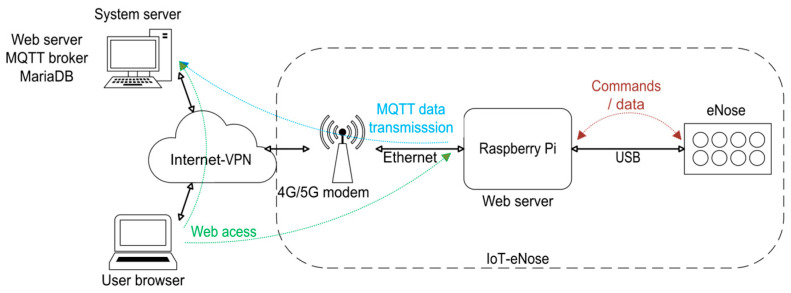
IoT-eNose components, connections and information flow.

The hardware elements included in the IoT-eNose are represented in Figure 4. The user interacts with the Raspberry Pi through a web server, whereas the communication between the Raspberry Pi and the eNose involves the transmission of commands and data through the USB link.

### 4.1. Raspberry-Pi Web Server

The web server permits the remote management of the eNose and encompasses all the essential functions required. This website must interact with the user, collect sensors’ samples from the eNose and save data in databases within the system server using the MQTT protocol. In this work, we utilized the Python programming language alongside the Flask micro framework [38] to perform these tasks. Flask is a lightweight and flexible micro framework for web development in Python, providing developers with complete control over the structure and functionality of their applications. It features a straightforward yet powerful Universal Resource Locator (URL) routing system and supports dynamic template rendering through Jinja2. Flask’s extensibility is a key strength, offering a wide range of extensions for functionalities such as web forms, websockets, database management and user authentication. Additionally, Flask includes built-in tools for development, debugging, and managing HTTP requests, cookies, and sessions. Due to its simplicity and versatility, Flask is particularly well-suited for creating medium-sized web applications, such as those used to control IoT device hardware.

**Figure 5:**
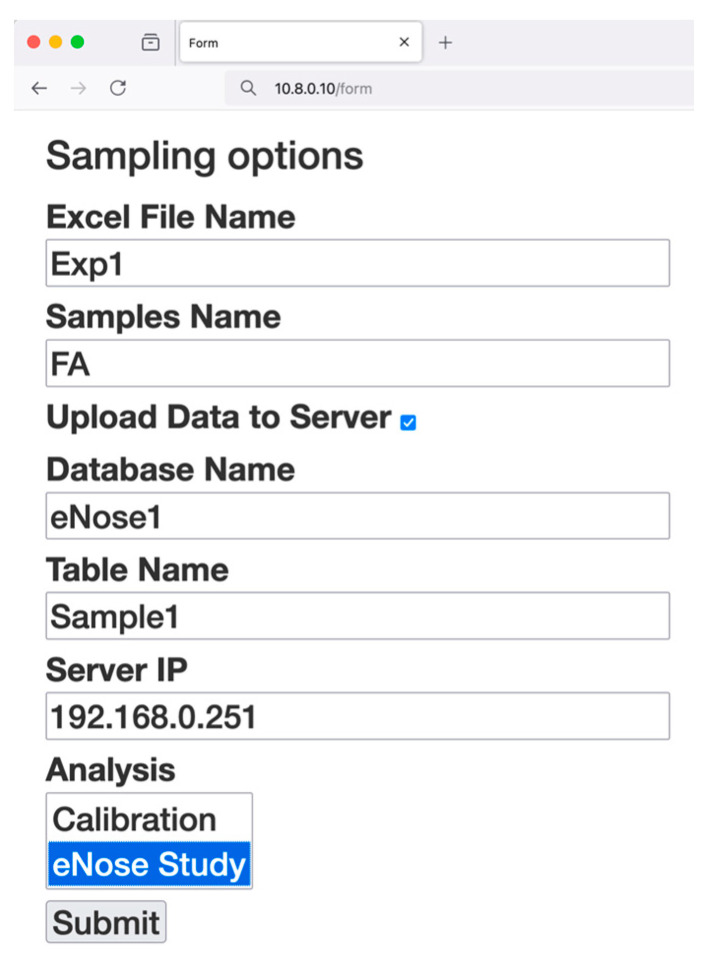
View of the user’s browser during the configuration of the sampling process.

Figure 5 shows the form implemented in Flask to permit the user to introduce the required information to start the sampling process of the eNose data. Users can save and download the data directly in an Excel format file. At the same time, they can select the option to upload the data to the system server providing its IP address, the name of the database and the table in this database where the data will be saved. Additionally, information about the type of sampling process is required, with two options possible: calibration or eNose study. These options allow us to perform a calibration of the integrated sensors or the data acquisition of a new sample.

**Figure 6:**
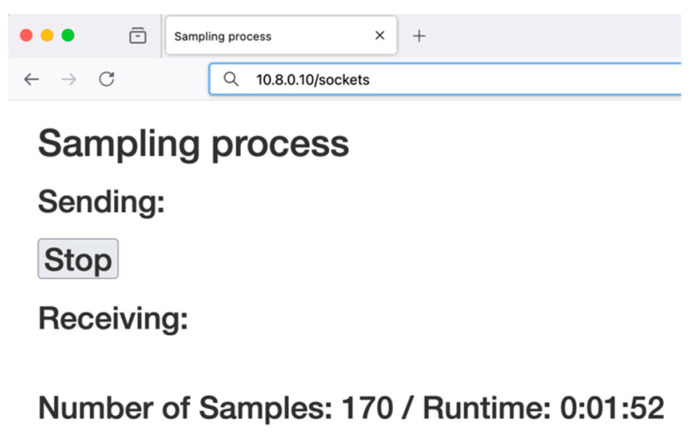
View of the user’s browser when receiving updates with the information about the sampling process.

Once the information in this form is submitted, the web application redirects the user to a different URL and sends the corresponding command to start the data sampling process though the USB link. The information of the data received in real-time is shown in the new URL. The HTML template of this route is defined and rendered using Jinja2, including a script for handling real-time updates using websockets. The template includes the socket script to establish a web-socket connection, which sends information about the number of samples received and the runtime of the data acquisition process. Figure 6 displays the user’s browser with the information received from the IoT-eNose. The web interface contains a stop button to conclude the sampling process.

### 4.2. USB Communication Between Raspberry Pi and eNose

As has been previously indicated, the Raspberry Pi communicates with the eNose via the USB. It sets up the serial port configuration by specifying the port (/dev/ttyUSB0) and the baud rate (115,200), then establishes a connection using the serial. Serial class was included in the Python V3 package pySerial. This component enables the transmission and reception of data through the USB link. After receiving the command to start the sampling process, the eNose collects the data from the eight sensors (Mq135, Mq2, Mq3, Mq4, Mq5, Mq6, Mq7, and Mq8) using a sampling period of 450 ms. The transmitted data structure comprises 25 Bytes and includes a sample counter, heating level of the sensors used in the sample, sensors’ data and humidity and temperature of the sample chamber [1]. Once the Raspberry Pi receives the USB packet, it updates the number of samples received and the experiment runtime in the user’s web interface, as shown in Figure 6. Then, it encapsulates all this information in a JSON format packet to retransmit the data to the system server using an MQTT client.

### 4.3. MQTT Client

The Raspberry Pi web server sends data in JSON format to an MQTT broker running on the system server. The MQTT broker makes this data available to all subscribers of that MQTT topic. In this implementation of the MQTT protocol, it operates over TCP, which inherently provides reliable packet transmission. However, configuring the QoS level to 0 does not guarantee message delivery or integrity at the MQTT broker. This level is designed for scenarios where some occasional message loss is acceptable, as is the case of the proposed IoT-eNose, since the loss of some samples does not significantly impact the data acquisition process. The code is based on the Paho library, which provides the functions required for connecting the client to the MQTT broker and for transmitting the collected data to the server. The topic of the published message is formed using the eNose ID, database name and the table name specified by the user. This topic structure allows the user to configure, through the sampling options of the Raspberry Pi web interface, the name of the database and the table that contains the data of the current experiment. Python functions executed for establishing the configuration of the MQTT client are:

mqttc = mqtt.Client()

mqttc.username_pw_set(login, pass)

After that, the transmission of one message is performed with the following functions:

mqttc.connect(Server_IP_address, port)

Topic = eNose_id + ‘/’ + Database + ‘/’ + Table

Qos = 0

mqttc.publish(Topic, Message_json, Qos)

mqttc.disconnect()

In the previous code, eNose_id, Database and Table are three strings combined to form the MQTT topic for this transmission. Additionally, QoS 0 was selected, indicating a best-effort approach without a delivery guarantee. The reliability achieved with this selection is discussed in the Results section.

## 5. System Server Architecture

The system server runs three main software components: (a) MariaDB database, (b) MQTT broker and client, (c) web server for Data management and visualization. Users can interact with the database to manage and visualize the data through a web interface. The implementation of these elements is detailed next.

### 5.1. MQTT Broker and Client

This computer runs the MQTT broker jointly with a MQTT client that is subscribed to the data messages received from the IoT-eNose. As in the previous case, these components are based on the Python MQTT Paho library. After the reception of the messages, the MQTT client parses the information and saves the data in a MariaDB database. This client is executed when a new message arrives, and it saves the data in a database and table specified in the topic used to publish the MQTT message. The MQTT client creates the database or the table if they are not found currently in the system server. The access of the MQTT client to the MariaDB database is performed with the Python package mysql.connector(), which is a Python library provided by Oracle that allows Python applications to interact with MySQL or MariaDB databases. It is part of the official MySQL Connector/Python, and it enables the execution of SQL queries, fetch results, and manage database connections seamlessly. The configuration of the MQTT client is performed with the following functions:

mqttc = mqtt.Client()

mqttc.on_message = on_message

This declares the function on_message as the handler for every new message that arrives at the MQTT broker with a topic matching the client’s subscription.

mqttc.username_pw_set(login, pass)

mqttc.connect(Server_IP_address, port)

These functions establish the connection to the MQTT broker running locally in the system server.

mqttc.subscribe(“#”, 0)

The subscription is set up with the topic “#” and QoS 0, meaning the broker sends messages to the client as soon as they are available, but without any delivery guarantee. Subscribing to topic “#” grants access to all topics, except those beginning with a “$”, which are typically control topics. The topic is used by the MQTT client to identify the database and the table where the data must be saved.

mqttc.loop_forever()

Finally, this method blocks the program and manages automatic reconnects, making it suitable for this case since the MQTT client only needs to react to incoming messages and save the information in the database.

### 5.2. Data Management and Visualization

The management of the MariaDB database is carried out though a web interface developed in Flask and Python running in the system server. Users can consult the existing databases and tables; they can download data from these tables in Excel files and delete them when they are no longer needed. The access to the database is based on the Python package mysql.connector() and the results of the queries are presented in the user’s browser.

Users can also require the graphical visualization of the data contained in a table. This operation is accomplished using the Bokeh library, which is integrated into the Flask web server. The graph is updated periodically with fresh data by means of the AjaxDataSource component, which is used to load data asynchronously from a web server into a Bokeh graph, enabling dynamic updates without requiring a full page reload. The AjaxDataSource continuously fetches updated data from the system server endpoints defined by URLs, ensuring that the latest information is displayed and allowing the supervision of the sampled data during an experiment. The Bokeh plot is embedded into the HTML page and displayed in the user’s browser, where it dynamically updates as new data is received. The JavaScript and CSS resources required for embedding the Bokeh plot are generated and rendered into an HTML template. Finally, the rendered HTML is returned to the user, providing a dynamic and visually updated page. This setup provides a seamless, real-time visualization experience for the user. All the Python programs developed to implement the IoT- eNose and the system server web interfaces can be found in the following Github repository [39].

## 6. Results

**Figure 7:**
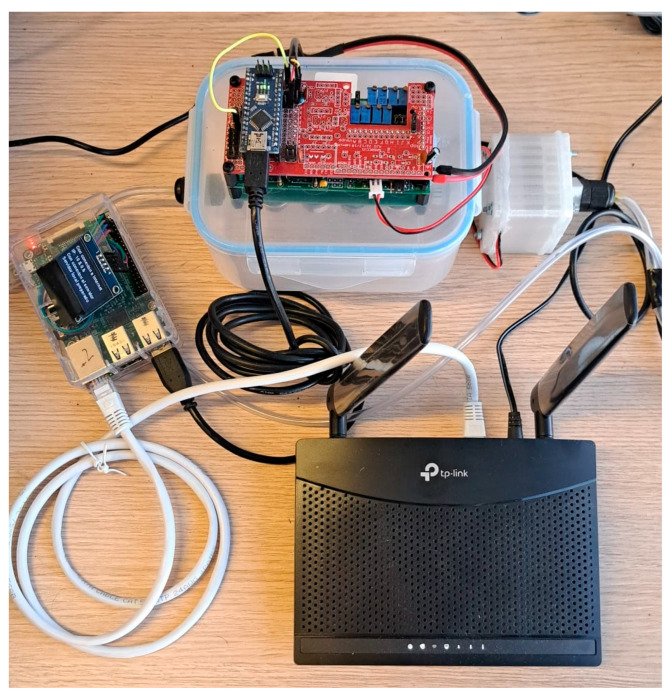
Interconnection of the IoT node: Raspberry Pi, electronic nose and 4G modem.

This section evaluates the performance of the system in a real-world environment. The system comprises an IoT-eNose, the system server and the router providing the VPN connection. The IoT-eNose responsible for data collection consists of a Raspberry Pi connected to an electronic nose through a USB port. The Raspberry Pi used is a Model 3 b+, featuring a Broadcom BCM2837B0 (Broadcom Inc., San Jose, CA, USA), Cortex-A53 (ARMv8) 64-bit SoC @ 1.4 GHz, and 1 GiB of SDRAM, whereas the eNose is the one specified in [1]. The Raspberry Pi connects to the Internet using a TP-LINK TL-MR101 4G modem (TP-Link Technologies Co., Ltd., Nanshan, Shenzhen, China) equipped with a SIM card of the Orange company, with both components linked by an Ethernet cable. Additionally, the Raspberry Pi runs an OpenVPN client to access a VPN hosted by a TP-LINK Archer AX53 router (TP-Link Technologies Co., Ltd., Nanshan, Shenzhen, China), which connects to the Internet using a static public IP address. This configuration establishes a secure private network to protect the Raspberry Pi’s web servers and the system server from unauthorized access. Figure 7 depicts the physical connection between the Raspberry Pi, the electronic nose, and the 4G modem. On the other hand, the system server setup is based on a personal computer with an Intel Core i7 processor (Intel Corporation, Santa Clara, CA, USA) and 32 GiB of DDR4 main memory (Micron Technology, Inc., Boise, ID, USA) running a Debian Linux distribution.

The experiments have focused on the assessment of the latency in the web server access, the reliability of the MTTQ transmission, resources utilized in the Raspberry Pi, bandwidth requirements and overall system functionality with data visualization. Next, each one of these items is explained.

### 6.1. Latency Assessment

**Figure 8:**
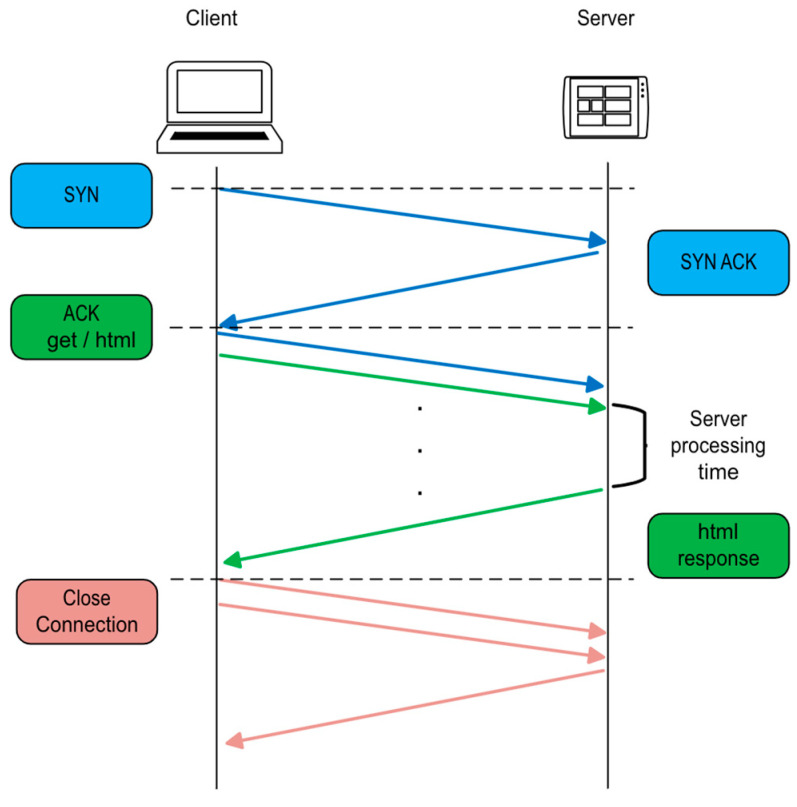
TCP packet exchange between client and server.

The first experimental analysis aims to measure the latency introduced when communication is established via a 4G modem. The measurement tries to determine the latency during the execution of HTTP GET and POST methods, which facilitate interaction between the user’s browser and the web server hosted on the Raspberry Pi in the IoT-eNose. The communication process adheres to the standard sequence defined by the HTTP protocol. It begins with the TCP three-way handshake: the client sends a SYN packet to initiate the connection, the server responds with a SYN-ACK, and the client finalizes the handshake with an ACK. Once the connection is established, the client sends an HTTP GET request specifying the desired resource using a URL. The server processes the request and returns an HTTP response, which includes a status line, headers, and the requested content (HTML page or data). After completing the data exchange, the TCP connection is terminated through a sequence of FIN and ACK packets exchanged by the client and server. Figure 8 illustrates this entire sequence of TCP packets and their interaction.

**Figure 9:**
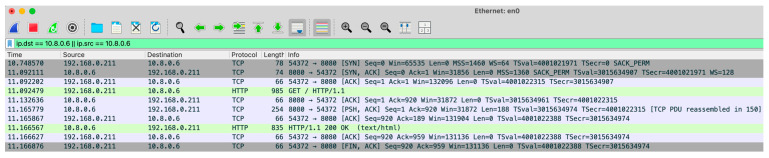
Wireshark capture of the sequence of packets during the client’s access to the Web server of the Raspberry Pi in the IoT node.

The latency for the entire sequence of packet exchanges is measured on the computer running the browser. To ensure the process was completed correctly, the TCP packet exchange between the computer and the Raspberry Pi was initially captured using the Wireshark V4.4.2 software. This capture verified the integrity and accuracy of the interaction. Figure 9 displays the sequence of exchanged packets, with the timestamp of each packet listed in the first column. These timestamps allow for precise measurement of the duration of the sequence, which is approximately 69 milliseconds.

**Figure 10:**
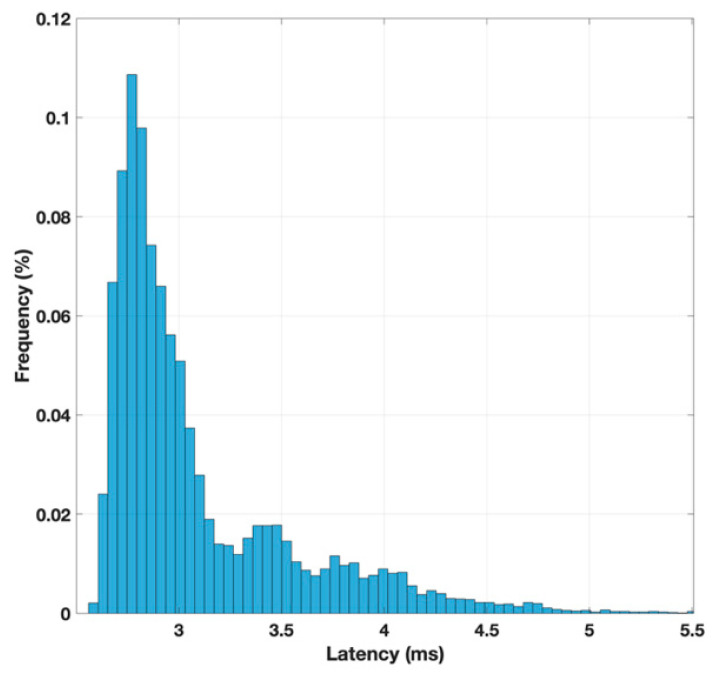
Latency histogram in the execution of an HTTP GET method with Ethernet connection.

**Figure 11:**
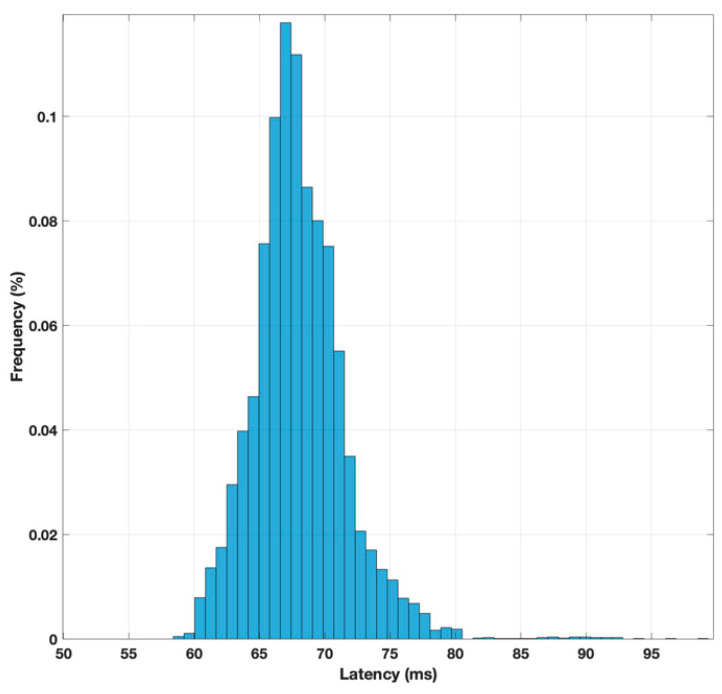
Latency histogram in the execution of an HTTP GET method with 4G connection.

To achieve statistically significant results, this experiment was repeated automatically ten thousand times to obtain a more reliable average value for the latency. Additionally, a second experiment was conducted to measure the latency when the Raspberry Pi was directly connected to the same router as the client computer via an Ethernet cable. The histograms representing the latency distributions for these two scenarios are shown in Figure 10 and Figure 11, respectively. The mean latency values for both cases were calculated, with a delay of 67 milliseconds for the 4G connection and 3 milliseconds for the one with the direct Ethernet connection. From these results, it can be concluded that the delay caused by the 4G connection significantly impacts the access time to the web server hosted on the Raspberry Pi. Specifically, the 4G connection increases the access latency by a factor of 20, highlighting the substantial influence of network delay on the server’s response time. This delay is a critical factor to consider in applications where the system is responsible for controlling elements that depend on a feedback loop with strict time constraints. In such scenarios, the introduced delay could disrupt the real-time response of the system, potentially leading to performance degradation or failure to meet the required timing for accurate operation.

Additionally, this latency can impact the web response time and the overall user experience when managing the IoT-eNose through the web interface. Keeping this time low enhances the user experience and reduces the server response time. The experimental results have shown that the web server in the Raspberry Pi can produce a rapid response to user requests. However, the use of cellular connection enlarges this response time due to the transmission latency, but even in this case, the time remains low enough to ensure a good user experience.

### 6.2. MQTT Messages Transmission Reliability

**Figure 12:**
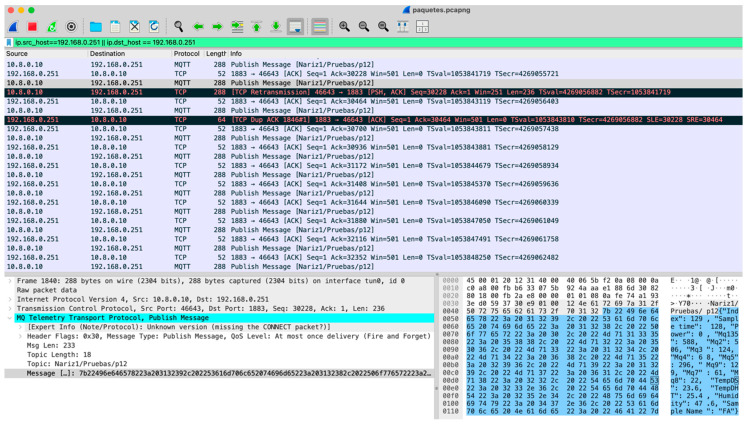
Wireshark capture with a TCP packet retransmission and ACK duplicate.

The goal of this experiment is to determine the system reliability during data transmission from the IoT-eNose to the system server using the MQTT protocol. As stated in the previous section, the QoS selected is 0, indicating that no reception guarantee is configured. This evaluation involved the transmission of 10,000 MQTT messages published from the IoT-eNose and their reception and storage in the system server database. To this end, an experiment with this number of data samples was carried out. During the test, a Wireshark session on the Raspberry Pi of the IoT-eNose was open to detect erroneous transmissions. As can be seen in Figure 12, the first detected error is due to a TCP retransmission, indicating that the sender did not receive an acknowledgment (ACK) for a previously sent packet within the expected time, prompting it to resend the packet. The second error detected indicates a duplicate ACK, which occurs when the receiver receives an out-of-order packet and is still waiting for a specific packet. This is a consequence of previous packet loss or delayed reception. After the completion of the test the percentage of TCP retransmissions detected was 0.5% of the total number of packets transmitted from the IoT-eNose to the system server with MQTT messages. Despite these packets’ losses and delays, TCP ensures packets are received in order and includes error checking and acknowledgment features, resulting in reliable packet transmission.

However, MQTT with QoS level 0 does not guarantee message delivery, acknowledgment, or retransmission, so data loss can still occur at this stage. Nevertheless, during the experiment conducted, no data loss was detected, and all the MQTT messages were correctly received by the subscriber running on the system server and saved in the MariaDB database. Thus, this experiment has corroborated that with a sampling rate of approximately two data messages transmitted per second, and using MQTT components based on the Python Paho library, reliable data transmission and storage can be accomplished with no data loss. This result confirms the robustness of the data collection process achieved in the proposed system with the MQTT protocol.

The network bandwidth used for transmitting the MQTT data sequence has also been measured using the Wireshark tool and it accounts for 4.6 kbps. If all the aggregated traffic is considered, including updates sent via websockets to the user’s browser, the total bandwidth rises to 9.4 kbps. Even though this bandwidth is quite low compared to the bandwidth provided by 4G/5G connections available with the modem used, which contributes to the correct transmission and storage of the collected data.

### 6.3. Resources Usage on the Raspberry Pi

**Figure 13:**
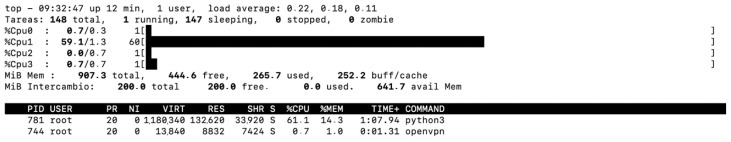
Capture of the top command output on the Raspberry Pi.

This evaluation tries to find out the resources utilized during the MQTT data transmission on the Raspberry Pi. The outcome of this evaluation determines if the Raspberry Pi fits the computational requirements imposed by the software components developed. The assessment measures the percentage of CPU time and main memory occupied by the Python program. The test was conducted employing the top Linux command, which provides the resources consumed by a program during its execution. Figure 13 shows the output of this command with the statistics for the python3 application and the OpenVPN client running. From these figures, it can be seen that the OpenVPN client does not represent a significant load in terms of CPU time and memory occupation. The percentage of memory used by the Python program is about 14% of the total memory of the system, which is not very high and leaves room for the execution of other applications. However, the Python program has an important impact on the CPU time since it requires around 60% of this time during the stage in which the MQTT protocol and the user’s browser updates via websockets are active. This represents an important load and can restrict the system’s capacity to execute other tasks. Nevertheless, as seen in Figure 13, this load is limited to only one of the four cores in the Raspberry Pi’s SoC, leaving the others practically free. Thus, the IoT-eNose still has the capability to execute other applications or tasks on the remaining free cores.

It is also important to note that the use of a VPN tunnel for secure communication did not introduce significant performance overhead in the system. As shown previously, the total bandwidth required for MQTT data transmission and websocket updates remained below 10 kbps. Given this low data rate, the encryption and tunneling operations performed by the OpenVPN client had a low impact on the system’s responsiveness and resource usage, confirming the suitability of VPN-based security even with resource-constrained IoT devices.

### 6.4. Data Visualization

The last experiment focuses on visualizing the data collected by the electronic nose in the user’s web browser. The Bokeh library integrated in the Python application running on the system server is utilized for dynamic data representation. In this setup, the system server provides access to data collected from the IoT-eNose that comprises the eight gas sensors, which are stored in the MariaDB database. Each sensor’s data can be accessed via eight distinct URLs hosted by the system server. When the user visits the main page, the browser first downloads the JavaScript code that performs the rendering of the graph, enabling the data visualization in the browser. Once the JavaScript is executed, the browser begins automatically retrieving and updating the data for each sensor channel. This is performed by sending requests to the corresponding web services associated with each sensor. The browser ensures that the data for each channel is continuously updated and the corresponding line in the graph is dynamically plotted to reflect real-time data changes.

**Figure 14:**
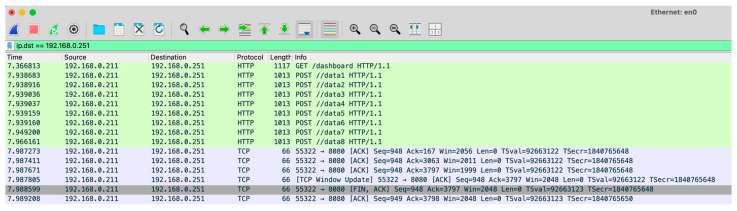
Wireshark capture of the sequence of packets during the client’s access to the web server of the system server.

A network traffic capture using Wireshark shows the sequence of URLs accessed by the browser to fetch the data, this capture is shown in Figure 14. In this experiment, the system server was directly connected to the router via an Ethernet cable to minimize external delays. The user computer requests data from all eight sensor channels, with each request retrieving the corresponding data from the database. To simplify the visualization of the web service access, many TCP data packets were omitted from the capture. This setup enables the calculation of the delay required to fetch the JavaScript code and sensor data needed for rendering the graph. In this specific configuration, with both the user computer and the system server directly connected to the router via Ethernet, the total time required for this process is approximately 622 milliseconds.

**Figure 15:**
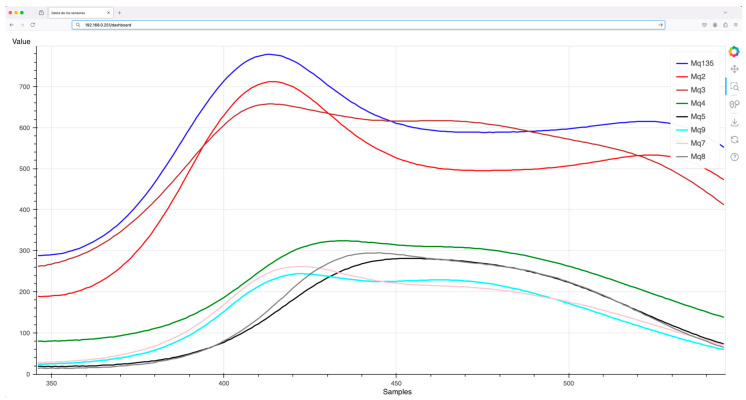
Visualization of the data from the gas sensors of the electronic nose.

Once the user’s browser receives all the necessary information, it can render the graph displaying the data and the values provided by each sensor. Figure 15 depicts the data graph as it appears in the user’s browser. The graph is designed to be updated automatically every 2 s, as specified by the parameters of the AjaxDataSource() function. This means the browser periodically fetches new data from the system server at this fixed interval. This continuous refresh mechanism ensures that the graph reflects real-time data as it is being collected by the IoT system and stored in the database, enabling dynamic and up-to-date visualization for the user. In addition, the measured delay in the retrieval of the data from the system server is lower than the update time configured in the AjaxDataSource() function, ensuring that this process can be performed efficiently.

The conducted experiments have confirmed the robustness of the system and the successful accomplishment of its intended functionality. The system provides a good response time, reliable and secure data transmission, and a low usage of IoT-eNose resources and bandwidth. This enables the real-time analysis of agricultural products in remote areas, ensuring timely and accurate data collection.

### 6.5. Application for Detecting Rot in Lemon Fruits

To evaluate the performance of the IoT-eNose system in a real-world agricultural setting, an experimental study was conducted using lemons provided by Citrus Gea Belmonte, S.L., a Spanish company specialized in citrus harvesting, and commercial distribution. The selected fruit belonged to the ‘Verna’ variety, known for its extended harvesting season and sensitivity to post-harvest conditions. All lemon samples were uniformly treated with 0.1% sodium benzoate (BS), a common preservative used to inhibit microbial growth and extend shelf life, as outlined in Table 2. The primary objective of the experiment was to assess the capability of the IoT-eNose to monitor changes in volatile organic compound (VOC) emissions over time, which are indicative of fruit freshness, ripeness, and potential spoilage.

To this end, the IoT-eNose system was deployed directly within the company’s facilities, enabling continuous and non-invasive monitoring of lemon batches under typical storage and handling conditions. Data acquisition was carried out over several weeks, capturing VOC profiles from multiple batches to allow for longitudinal analysis. This setup provided a realistic environment to evaluate the system’s robustness and sensitivity to temporal variations in VOC emissions, which may be influenced by pre-harvest treatments, storage parameters, and environmental factors. By integrating sensor data with time-series analysis, the experiment aimed to uncover patterns that could support early detection of quality degradation.

The organization of the fruits for this study was meticulously employing a randomized and homogeneous distribution approach. Each experimental condition consisted of three distinct lots, with each lot containing 10 lemons. The distribution strategy ensured that control lemons were grouped together, while two additional lots underwent field treatments involving sodium benzoate (BS) additive applications, specifically labeled as BS0.1 × 3 and BS0.1 × 4 passes. To maintain consistency and traceability, all lots were meticulously placed in IFCO boxes, each box appropriately labeled to indicate the respective treatments applied.

Measurement sessions using the IoT-eNose were systematically scheduled for 4 weeks. The first batch (CNT) underwent analysis every Monday across weeks 1 through 4. Similarly, the second batch (BS0.1 × 3) was analyzed every Tuesday during the same weeks, while the third batch (BS0.1 × 4) underwent analysis every Wednesday. This staggered schedule allowed for a comprehensive month-long monitoring period for each lot, enabling thorough evaluation of rot development through the IoT-eNose analyses. Such a systematic approach facilitated the detection of volatile organic compound patterns associated with fruit decay progression over time. It is pertinent to note that when the fruits were not undergoing analysis, they were stored in cold rooms maintained at a controlled temperature of 8 °C and relative humidity ranging between 80% and 85%. This storage condition was meticulously managed to prevent cold damage and maintain fruit quality until analysis, ensuring that environmental conditions did not compromise the integrity of the samples during the experimental period. This experimental setup and logistical framework not only ensured the systematic evaluation of rot development under controlled conditions but also underscored the importance of precise scheduling and environmental control in agricultural research. The use of IoT-eNose technology in conjunction with standardized storage protocols represents a significant advancement in fruit quality assessment, offering insights that can inform decision-making processes aimed at optimizing crop management practices and post-harvest handling techniques.

Principal Component Analysis (PCA) was employed to analyze and group the samples based on the collected data. The sample distribution was intentionally designed to reflect both within-group variability and treatment contrast, using a randomized and balanced block structure (3 lots of 10 lemons per treatment), ensuring replicability and statistical robustness. However, we will now include a justification matrix linking the biological variability of lemon VOC emissions with sensor response dynamics. Initially, the data were structured into a matrix format, followed by normalization to ensure each variable contributed equally to the analysis. This step was crucial to prevent variables with larger scales from dominating the principal components (PCs) solely based on their magnitude. The matrix was then decomposed into principal components (PCs), which are new variables that capture the maximum variance in the data. These components were ordered such that the first PC explains the most variance, followed by the second PC, and so on.

**Figure 16:**
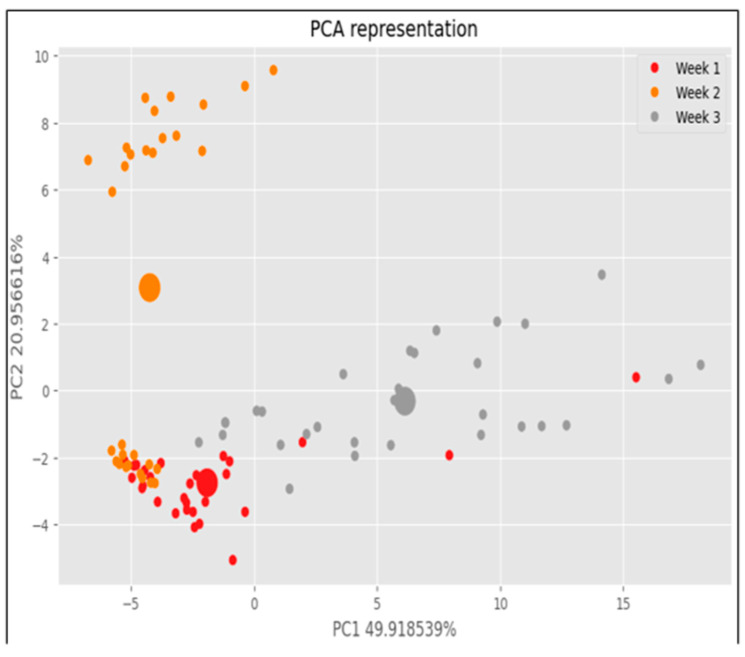
Sample distribution depending on the week of analysis.

The dataset used for PCA consisted of 90 data points, corresponding to measurements obtained from fruits using the IoT-eNose prototype. Figure 16 visually represents the results of the PCA performed on these data points. The analysis aimed to explore how the samples clustered relative to the week of analysis, providing insights into temporal variations or trends. Each sample is visually depicted as a point in the plot, with larger points indicating cluster centroids that summarize the groupings of data points. In Figure 16, samples analyzed during week 1 are depicted in red, primarily clustering in the lower left part of the plot. This clustering suggests that samples from week 1 share similar characteristics, possibly related to their composition or condition during measurement. In contrast, samples from week 2 are shown in orange and tend to cluster in the upper left portion of the plot, indicating a distinct grouping from week 1. Samples from week 3 are represented in gray and exhibit a more scattered distribution across the right side of the plot, suggesting greater variability or diversity among these samples compared to weeks 1 and 2. Overall, the PCA provided a structured approach to discerning patterns and grouping samples based on their characteristics derived from the IoT-eNose measurements. By focusing on specific phases and cycles of data collection, we aimed to extract meaningful insights that could inform further research or practical applications in quality assessment or classification tasks related to fruit analysis.

**Figure 17:**
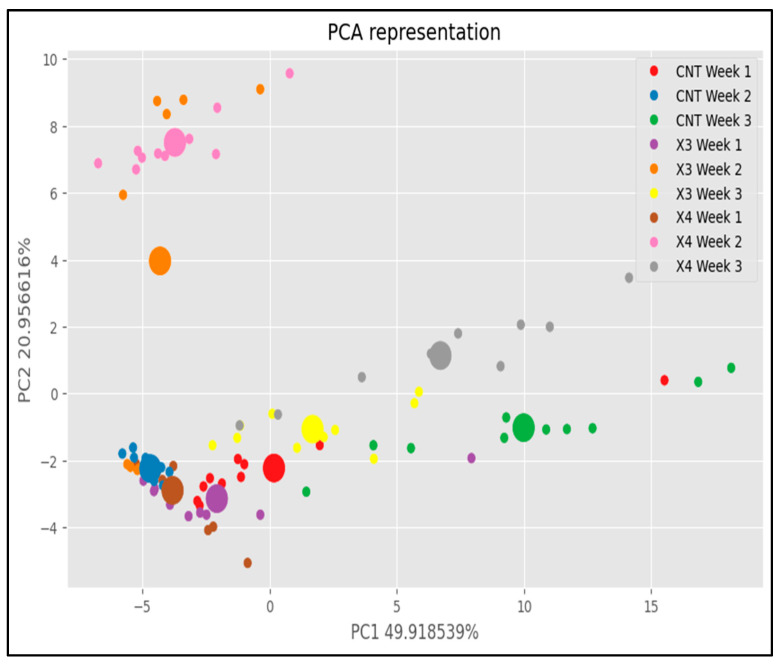
Sample distribution depending on the week of the analysis and number of applied passes in the field.

On the other hand, Figure 17 provides a detailed illustration of how the data are grouped based on both the week of analysis and the specific treatments applied. In this experimental setup, there were three distinct treatments and a span of three weeks over which the analyses were conducted, resulting in a total of nine distinct groups. The primary objective of this analysis was to elucidate the relationship between the applied treatments and the incidence of fruit rot, thereby evaluating the efficacy of each treatment method. Upon closer examination of Figure 17, several notable patterns emerged. Initially, during week 1 of the analysis, it was observed that samples from all three treatments exhibited a tendency to cluster together in the lower left part of the plot. This clustering was particularly evident among control samples, which formed a distinct cluster within this region. Additionally, some samples from the BS0.5 × 3 treatment group during week 2 also showed a tendency to join this cluster.

The distinct grouping of samples according to treatment and week highlights potential correlations between treatment application and the observed outcomes related to fruit rot. Each treatment’s efficacy in mitigating or influencing the development of fruit rot can be inferred from the clustering patterns observed in Figure 17. By visually mapping these clusters, it becomes possible to assess how each treatment performed across different time points, offering insights into their effectiveness under varying conditions. Moreover, beyond mere visual observation, statistical analysis can further validate these clustering patterns and provide quantitative measures of treatment efficacy. Techniques such as analysis of variance (ANOVA) or clustering algorithms can be employed to assess the significance of these groupings and determine whether the observed differences are statistically significant. Furthermore, understanding the dynamics of how treatments interact with temporal factors such as the progression of weeks is crucial. For instance, the consistent clustering of control samples in week 1 suggests a baseline level of fruit rot incidence before any treatment effects are applied. In contrast, the variability in clustering observed in subsequent weeks, particularly with the BS0.5 × 3 treatment group, may indicate varying levels of treatment effectiveness over time or in response to changing environmental conditions.

In conclusion, Figure 17 serves as a visual representation of the complex relationships between treatments, weeks of analysis, and the incidence of fruit rot. It underscores the importance of systematic analysis and data visualization in agricultural research, offering insights that can inform future experimental designs and agricultural practices aimed at optimizing fruit quality and yield. Further exploration of these patterns through advanced statistical methods will contribute to a more comprehensive understanding of treatment impacts on fruit health and overall crop management strategies.

**Figure 18:**
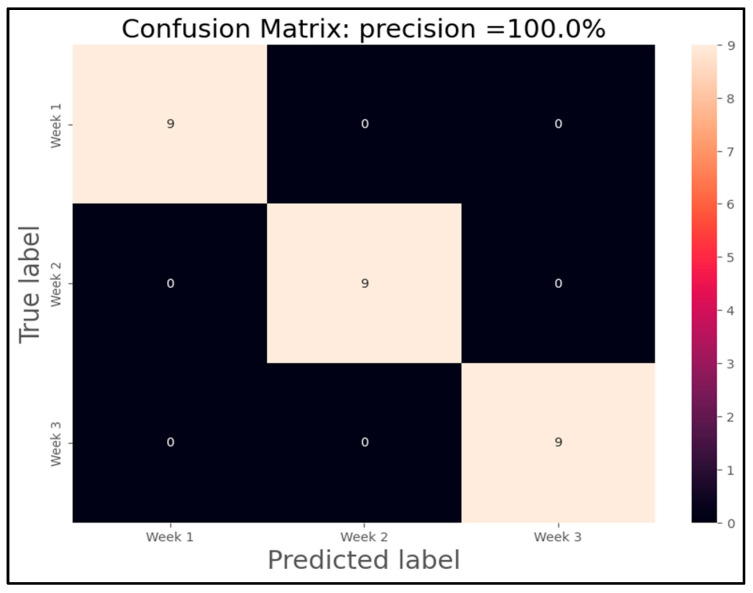
Confusion matrix of SVM model for sample classification for weeks.

**Figure 19:**
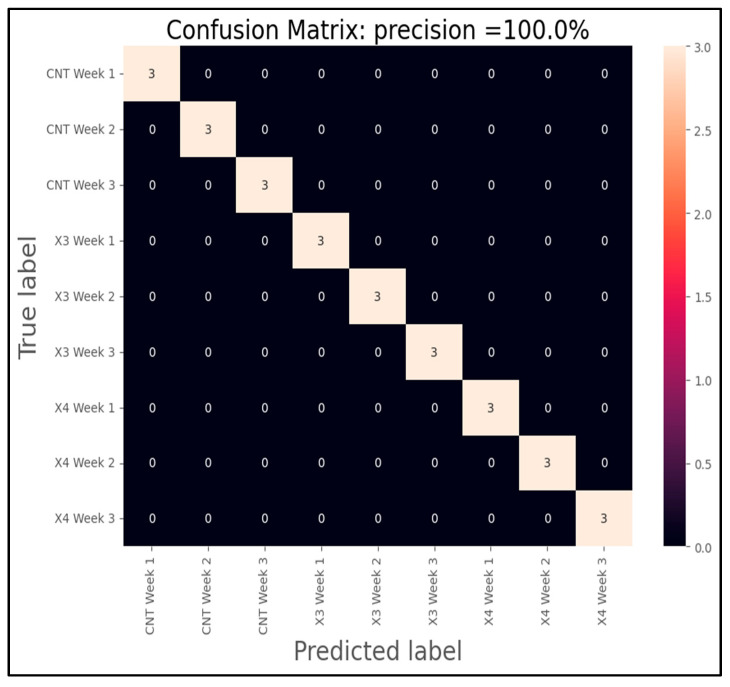
Confusion matrix of SVM model for sample classification for weeks and applied type treatment.

Finally, the culmination of our analysis involved the training of two Support Vector Machine (SVM) models designed to predict distinct scenarios based on the data collected. These models were developed using k-means clustering as part of their preprocessing step to enhance classification accuracy. Figure 18 visually presents the outcomes of the SVM model tailored to classify lemons according to the week of analysis, while Figure 19 demonstrates the classification results considering both the week of analysis and the specific treatment applied to the lemons.

In the experimental setup, the dataset encompassed comprehensive measurements and observations over multiple weeks, with three distinct treatments under scrutiny. This comprehensive approach allowed for a nuanced evaluation of how temporal factors and treatment variations influence the classification outcomes. Upon rigorous training and validation, both SVM models exhibited exceptional performance in their respective classification tasks. Notably, the model depicted in Figure 18 achieved a remarkable 100% accuracy in classifying lemons according to their respective weeks of analysis. This flawless accuracy underscores the robustness of the SVM algorithm in capturing the temporal patterns inherent in the dataset. In contrast, Figure 19 showcases the results of the SVM model tasked with classifying lemons based on both the week of analysis and the specific treatment applied. Despite the added complexity of predicting outcomes influenced by treatment variations, this model also achieved a notable 100% accuracy in its classification task. This achievement highlights the efficacy of SVM models in handling multifaceted datasets and discerning subtle variations in classification patterns across different experimental conditions.

However, it is essential to note the role of k-means clustering in enhancing the initial classification accuracy. In the first scenario, where classification was based solely on the week of analysis, the SVM model achieved 100% accuracy. In contrast, when k-means clustering was employed as a preprocessing step to refine the classification boundaries, the accuracy slightly decreased to 92%. This slight reduction suggests that while k-means clustering effectively aids in grouping similar data points, there may still be inherent variability that impacts classification outcomes. Similarly, in the second scenario where classification considered both the week of analysis and treatment variables, the SVM model initially achieved 100% accuracy. However, with the inclusion of k-means clustering as a preprocessing step, the classification accuracy notably decreased to 34%. This observation reveals an over-simplification of the input space, leading to a loss of critical variance required for effective neural network learning. This significant reduction highlights the inherent complexity of classifying data points when multiple variables are considered simultaneously, necessitating further investigation into refining the clustering and classification methodologies. This reinforces the conclusion that preprocessing methods must preserve VOC signature fidelity for IoT-eNose applications. Despite statistical evaluation, the technical validation of the IoT-eNose was demonstrated by its ability to distinguish decay stages and treatment effects in real-time under field-mimicking conditions.

The comprehensive evaluation of these SVM models and their associated preprocessing techniques not only underscores their potential in accurately predicting experimental outcomes but also emphasizes the importance of robust data preprocessing methods in optimizing classification performance. Moving forward, continued research efforts will focus on refining clustering algorithms and enhancing SVM model capabilities to handle increasingly complex datasets and diverse experimental conditions.

In conclusion, Figure 18 and Figure 19 provide compelling visual representations of the SVM models’ capabilities in classifying lemons based on temporal and treatment-related factors. The achieved accuracies demonstrate the potential of machine learning algorithms in agricultural research settings, where precise classification of experimental outcomes can significantly impact decision-making processes and enhance agricultural practices for improved yield and quality outcomes, specifically rot monitoring.

## 7. Conclusions

This article presents the design and deployment of a web-based IoT-eNose for remote agricultural product analysis. This device includes a Raspberry Pi to perform real-time data collection and transmission and runs a web server that allows interaction with the final user via a browser. This facilitates system deployment because it is not necessary to develop a user-specific application. The IoT-eNose is equipped with a 4G/5G cellular modem that allows Internet access at locations where the product to be tested is located and samples data from eight gas sensors. The system also contains a server where the data is transmitted using the MQTT protocol and saved in a MariaDB database. To secure system operation, a router providing a VPN server is included. The integration of OpenVPN for secure communication ensures that the IoT-eNose and the system server are protected from unauthorized access, a critical aspect for deploying IoT systems in remote or unsecured lo-cations. Additionally, the system server provides web services allowing access to this data for real-time visualization using a browser interface. This architecture enables remote monitoring with secure and real-time data retrieval and visualization.

The system’s performance has been evaluated in a real-world setting, with an emphasis on understanding the impact of network latency, especially when utilizing a 4G/5G connection for communication. The system successfully facilitated remote data collection and visualization, leveraging IoT technologies to monitor agricultural products in real-time. The experiments revealed a significant performance discrepancy between a 4G connection and a direct Ethernet connection. The 4G network increased latency by a factor of 20, which has critical implications for applications with strict real-time requirements. However, this latency delay is still acceptable since it does not significantly degrade the response time during access to the IoT-eNose web interface.

The resources required to deploy and run the web server on the Raspberry Pi have been measured. This evaluation indicates that the resources occupied by the Flask server remain low, even with continuous transmissions of MQTT data messages and updates sent to the user’s browser via web-sockets. The total amount of memory and CPU time used is less than a fourth of the Raspberry Pi’s total capacity, allowing additional tasks and applications to run if necessary. The total bandwidth used in the transmission of IoT-eNose information and data is also quite low, ensuring the viability of these transmissions using cellular technology. In terms of data integrity and reliability in transmission and storage, the system performs correctly without any data loss, given the established information and sampling period for the developed IoT-eNose. The TCP capability to detect packet delays and losses, along with the robustness of the MQTT publisher/subscriber procedure, even with a QoS of 0, has enabled complete data collection of large datasets. The use of the Bokeh library to visualize data in the browser demonstrated the system’s ability to deliver dynamic, real-time insights from the data collected.

Based on the results obtained in this study, it can be inferred that the IoT-eNose demonstrates significant capability in differentiating lemon samples analyzed across three distinct weeks, effectively correlating with the fruit’s decay progression. The potential of the eNose as a monitoring tool for lemon decay is considerable. Its ability to detect and differentiate between subtle changes in fruit aroma profiles associated with decay progression offers promising applications in agricultural settings. Utilizing the IoT-eNose, farmers and producers could potentially optimize storage conditions, reduce post-harvest losses, and ensure higher quality standards for harvested citrus fruits. These findings underscore the transformative potential of this technology in advancing precision agriculture and enhancing post-harvest fruit quality management.

This work opens several promising opportunities for future research and technological innovation. One key direction involves the integration of advanced machine learning (ML) and deep learning (DL) techniques to enhance the analytical capabilities of the IoT-eNose system. By leveraging these methods, it will be possible to automatically classify the quality and condition of various agro-food products based on their VOC profiles. The goal is to embed intelligence within the system, enabling real-time detection of changes in product samples and supporting proactive decision-making in agricultural and food supply chains. Due to the computational demands of these algorithms, they will be deployed on the system’s central server, ensuring efficient processing while maintaining real-time accessibility for end users through connected interfaces.

Another critical area for future exploration is the scalability and interoperability of the system architecture. As the deployment of IoT-eNose units expands, ensuring robust and reliable communication via MQTT protocols, as well as efficient data storage and integration, becomes increasingly important. To address these challenges, dynamic resource allocation on the system server will be essential. This includes the ability to scale computing power and storage capacity in response to real-time demand, thereby minimizing latency, optimizing performance, and controlling operational costs. In this context, the adoption of cloud computing technologies offers a flexible and scalable solution for managing infrastructure resources, facilitating seamless integration of multiple devices, and supporting the long-term sustainability of the platform.

## Figures and Tables

**Table 1:** Summary of applications of MOS-based eNoses.

Application Area	Description	References
Healthcare	Non-invasive disease diagnosis via breath analysis (e.g., cancer, respiratory diseases)	[13,14,15]
Food and Beverage	Quality and freshness control, detection of contaminants/adulteration in various foods	[1,16,17,25,26]
Agriculture	Early detection of crop diseases and pests by monitoring emitted VOCs	[18,19,20,27]
Environmental Monitoring	Detection of pollutants, odor control, and process monitoring in air, water, and waste management	[21,22,23]

**Table 2:** Employed treatments in the fruits.

Variety	Treatment	Concentration	Abbreviation
Verna	Control	-	CNT
Verna	Sodic Benzoate	0.1 × 3 passes	BS0.1 × 3 passes
Verna	Sodic Benzoate	0.1 × 4 passes	BS0.1 × 4 passes

## Data Availability

The raw data supporting the conclusions of this article will be made available by the authors on request.
